# Dichloroacetate enhances the antitumor effect of pirarubicin via regulating the ROS-JNK signaling pathway in liver cancer cells

**DOI:** 10.20517/cdr.2020.32

**Published:** 2020-09-04

**Authors:** Xiao-Jing Yan, Peng Xie, Xu-Fang Dai, Ling-Xi Chen, Liang-Bo Sun, Tao Li, Wen-Hui He, Zhi-Zhen Xu, Gang Huang, Feng-Tian He, Ji-Qin Lian

**Affiliations:** ^1^Department of Biochemistry and Molecular Biology, Army Medical University, Chongqing 400038, China.; ^2^Department of Educational Science College, Chongqing Normal University, Chongqing 400038, China.; ^*^Equal contribution to this work.

**Keywords:** Dichloroacetate, c-Jun N-terminal kinase, liver cancer cells, pirarubicin, reactive oxygen species

## Abstract

**Aim**: Liver cancer is one of the most common malignancies and has a high recurrence rate. However, current treatment strategies do not achieve satisfactory outcomes in the clinic. To explore a new strategy to enhance the effectiveness of chemotherapy in liver cancer, we investigated whether dichloroacetate (DCA) could enhance the sensitivity of liver cancer cells to pirarubicin (THP).

**Methods**: Liver cancer cells were treated with DCA alone, THP alone, or DCA and THP combined. Cell viability was determined by the CCK-8 assay. Cell apoptosis was analyzed by flow cytometer. Reactive oxygen species (ROS) were detected using a CM-H2DCFDA fluorescence probe. Protein levels were identified by immunoblotting.

**Results**: The results revealed that DCA significantly enhanced the antitumor effect of THP in liver cancer cells. Changes in morphology and adherence ability were observed, as well as decreased cell viability. The results of flow cytometry showed that the combination of THP and DCA significantly increased apoptosis of liver cancer cells. Moreover, compared with THP alone, combination treatment with DCA significantly increased THP-triggered ROS generation in liver cancer cells. The antioxidant N-acetyl-L-cysteine reversed the synergistic effect of DCA and THP on ROS generation, cell viability and apoptosis. Furthermore, phosphorylation of c-Jun N-terminal kinase (JNK) was significantly increased in the DCA and THP combination group. The effects of DCA and THP on cell viability and apoptosis were inhibited by the JNK inhibitor SP600125.

**Conclusion**: The results obtained in the present study indicated that DCA enhanced the antitumor effect of THP in liver cancer cells via regulating the ROS-JNK signaling pathway.

## Introduction

Liver cancer is the third leading cause of cancer mortality worldwide^[1]^. Surgical resection, thermal ablation and liver transplantation are current treatments for early-stage liver cancer^[2]^. However, liver cancer is often diagnosed at an advanced stage and is associated with poor prognosis. Pirarubicin hydrochloride (THP), a novel anthracycline anticancer drug, has been widely used in transcatheter arterial chemoembolization (TACE) for the treatment of primary liver cancer^[3,4]^. THP exhibits a wide antineoplastic spectrum, strong antitumor activity and minimal toxicity^[5]^. However, 50% patients with liver cancer do not respond to initial TACE, due to tumor response and chemotherapeutic resistance^[6,7]^. Therefore, the identification of novel drug combinations may overcome chemoresistance and improve patient outcomes.

Dichloroacetate (DCA), a synthetic pyruvate dehydrogenase kinase (PDK) inhibitor, has been shown to induce cell death in a variety of cancer cells, including lung, prostate, breast, medullary thyroid cancer cells and myeloma and glioblastoma multiforme cells^[8,9]^. PDK regulates cellular metabolism by inhibiting pyruvate dehydrogenase (PDH) and DCA reverses the glycolytic phenotype and reduces lactic acid^[9]^. A limited number of studies on the combination of DCA and cytotoxic drugs or radiation therapy are currently available. Kan *et al.*^[10]^ studied the effect of DCA in combination with curcumin in liver cancer. In addition, Shen *et al.*^[11]^ demonstrated that DCA increases the sensitivity of liver cancer cells to sorafenib. However, to the best of our knowledge, whether DCA enhances the cytotoxic effect of THP has not been previously reported. Therefore, the aim of the present study was to investigate the synergistic effect of DCA and THP in liver cancer cells, and the related molecular mechanisms as well to provide a theoretical basis for the clinical treatment of liver cancer.

## Methods

### Cell culture

The human liver cancer cell lines Hep3B and Huh7 were purchased from the American Type Culture Collection and cultured in DMEM (GE Healthcare Life Sciences, Little Chalfont, Buckinghamshire, UK) supplemented with 10% FBS (GE Healthcare Life Sciences), streptomycin (100 μg/mL) and penicillin (100 U/mL). The two lines were originally tested by ATCC and passaged < 6 months in the laboratory.

### Reagents and antibodies

Sodium dichloroacetate (cat. no. HY-Y0445A) and pirarubicin hydrochloride (cat. no. HY-13725A) were purchased from MedChemExpress (Monmouth Junction, NJ, USA). N-Acetyl-L-cysteine (cat. no. A7250) and SP600125 (cat. no. S5567) were purchased from Sigma-Aldrich (Merck KGaA, St. Louis, MO, USA). The Annexin V-FITC Apoptosis Detection kit (cat. no. C1062) was purchased from Beyotime Institute of Biotechnology (Shanghai, China). The Cell Counting Kit-8 (CCK-8; cat. no. CK04) was purchased from Dojindo Molecular Technologies (Kumamoto, Japan). Antibodies against poly (ADP-ribose) polymerase 1 (PARP, cat. no. 9532), ERK (cat. no. 4695), phosphorylated (p)-ERK (cat. no. 4370), AKT (cat. no. 4685), p-AKT (cat. no. 9611), JNK (cat. no. 9252) and p-JNK (cat. no. 9255) were obtained from Cell Signaling Technology (Danvers, MA, USA). Antibodies against β-tubulin (cat. no. sc-166729) were obtained from Santa Cruz Biotechnology (Santa Cruz, CA, USA).

### Cytotoxicity assay

Cells were seeded at a density of ~2,000 cells per well in 96-well plates and cultured overnight. The cells were then treated with THP (0, 150, 300, 600 or 900 nmol/L) or DCA (0, 10, 20, 40 or 60 mmol/L) or THP (300 nmol/L) combined with DCA (20 mmol/L) for 24 h. Control cells were treated with dimethyl sulfoxide (DMSO). The culture medium without cells was used as the blank control. Subsequently, the cells and medium were incubated with CCK-8 reagent for 2 h in the dark according to the manufacturer’s protocol. Finally, absorbance was measured at a wavelength of 450 nm using a microplate reader. Viability was determined as follows: viability (%) = [1 - (At-Ab)/(Ac-Ab)] × 100^[12]^, where At, Ab and Ac represent absorbance values of treatment, blank and control, respectively. The experiments were performed in triplicate. To determine whether the combination of DCA and THP showed a synergistic effect, the combination index (CI) was analyzed according to the method of Chou and Talaly^[13]^. CI values of < 1, 1 and > 1 indicated synergistic, additive, and antagonistic effects, respectively.

### Western blotting

Cells were harvested and whole-cell lysates were prepared. Protein concentration was measured using a bicinchoninic acid protein assay kit (cat. no. P0012, Beyotime Institute of Biotechnology). Subsequently, proteins were separated by 10% or 12% sodium dodecyl sulfate polyacrylamide gel electrophoresis and were electrophoretically transferred to a polyvinylidene difluoride membrane (Bio-Rad Corporation, Hercules, CA, USA). The membrane was then incubated with primary antibodies, followed by horseradish peroxidase-labeled secondary antibodies. Tublin was used as a protein loading control.

### Measurement of intracellular reactive oxygen species

Cells were seeded in 24-well plates and treated with THP (300 nmol/L) or DCA (20 mmol/L) or THP (300 nmol/L) combined with DCA (20 mmol/L) for 24 h. Control cells were treated with DMSO. Intracellular reactive oxygen species (ROS) were assessed with the cell-permeable dye CM-H2DCFDA (cat. no. C6827; ThermoFisher Scientific (Waltham, MA, USA), 2 mmol/L in DMSO as a stock solution). In brief, the treated cells were washed three times with PBS and then incubated with 2 µmol/L CM-H2DCFDA fluorescence probe for 25 min at room temperature. After washing twice with PBS, the cells were observed under a fluorescence microscope (IX81, LYMPUS) and the signal intensity was quantified by flow cytometry (CytoFLEX, Beckman Coulter, Miami, FL, USA) at an excitation wavelength of 488 nm and an emission wavelength of 525 nm. The ROS level of the control group was defined as “1” and the relative ROS level of the experimental groups was defined as “fold change” compared with the control group.

### Detection of apoptosis

Apoptosis was evaluated using the Annexin V-FITC apoptosis detection kit (cat. no. APOAF-50TST, Sigma-Aldrich) according to the manufacturer’s instructions. Briefly, the treated cells were harvested and incubated with Annexin V-FITC and propidium iodide (PI) at room temperature for 10 min. Subsequently, the samples were analyzed with a flow cytometer (CytoFLEX, Beckman Coulter)^[14]^. The treated cells were also stained with Hoechst 33258 to observe the nuclear morphology. Cells were fixed with methanolic acetic acid for 10 min and stained with Hoechst 33258 (10 μg/mL; Beyotime Institute of Biotechnology) for 10 min at room temperature in the dark. The cells were then washed twice with PBS, observed under a fluorescence microscope and imaged. Apoptotic cells were identified on the basis of nuclear morphological changes.

### Statistical analysis

Statistical analyses were carried out using GraphPad 6.0 (GraphPad Software, Inc., San Diego, CA) and SPSS 20.0 (SPSS Inc., Chicago, IL) software. The data are presented as the mean ± SD. Two-way ANOVA was used to analyze the variance of the different groups. *P* < 0.05 was considered to indicate a statistically significant difference.

## Results

### DCA enhances the antitumor effect of THP in liver cancer cells

**Figure 1:**
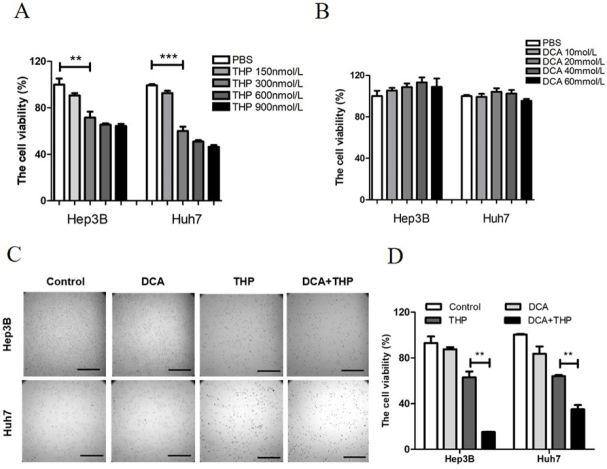
DCA enhances the antitumor effect of THP in liver cancer cells. A: Hep3B and Huh7 cells were treated with THP at different concentrations (0, 150, 300, 600 or 900 nmol/L) for 24 h, and cell viability was then determined with a CCK-8 kit; B: Hep3B and Huh7 cells were treated with DCA at different concentrations (0, 10, 20, 40 or 60 mmol/L) for 24 h, and cell viability was then determined with a CCK-8 kit. The experiment is representative of at least three independent experiments. ^**^P < 0.01; C, D: the liver cancer cell lines Hep3B and Huh7 were treated with DCA (20 mmol/L), THP (300 nmol/L) or a combination of DCA and THP for 24 h. Cell morphology was then observed under a microscope (C) and cell viability was determined with a CCK-8 kit (D). Experiments were repeated at least three times. ^**^P < 0.01. DCA: dichloroacetate; THP: pirarubicin

The present study investigated the effect of THP and DCA on the viability of liver cancer cells using the CCK-8 assay. This assay is a tool for studying the induction and inhibition of cell proliferation *in vitro*. It is a very convenient assay, which uses a highly water-soluble tetrazolium salt. WST-8 [2-(2-methoxy-4-nitrophenyl)-3-(4-nitrophenyl)-5-(2,4-disulfophenyl)-2H-tetrazolium monosodium salt], which produces a water-soluble formazan dye upon reduction in the presence of an electron mediator. The CCK-8 assay is a sensitive colorimetric assay for the determination of the number of viable cells in cell proliferation and cytotoxicity assays. As shown in Figure 1A and B, treatment with THP alone lowered cell viability in a dose-dependent manner, while DCA alone did not affect cell viability. As 300 nmol/L THP had a significant effect on cell viability at a relatively low dose, this dose was selected for further experimentation. DCA was used at a dose of 20 mmol/L. Preliminary data showed that 300 nmol/L THP and 20 mmol/L DCA alone did not significantly affect the morphology and adherence ability of liver cancer cells [Figure 1C]. However, the combination of THP and DCA significantly decreased cell number and cell adherence ability and altered cell morphology [Figure 1C]. Furthermore, compared with THP or DCA alone, the combination of THP and DCA significantly reduced cell viability in liver cancer cells [Figure 1D]. To assess whether the combined effect was synergistic or additive, the combination index (CI) value was calculated, where CI less than 1 would be considered synergistic for the combination treatment^[13]^. The combination of DCA and THP resulted in a synergistic effect (CI = 0.288 in Hep3B cells and 0.580 in Huh7 cells). Meanwhile, treatment with THP alone, DCA alone or the combination of THP and DCA did not affect cell viability or adherence ability in normal hepatic cells [Supplementary Figure 1A and B]. These results indicated that DCA significantly enhanced the killing effect of THP in liver cancer cells.

### DCA promotes THP-induced apoptosis in liver cancer cells

**Figure 2:**
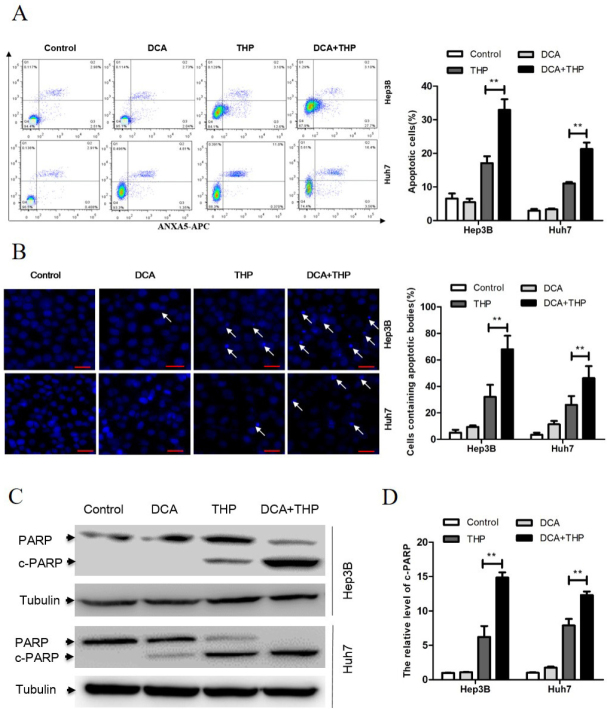
DCA promotes THP-induced apoptosis in liver cancer cells. Hep3B and Huh7 cells were treated with DCA (20 mmol/L), THP (300 nmol/L) or a combination of DCA and THP for 24 h. A: cells were processed for flow cytometry analysis, and the percentage of apoptotic cells was calculated as the percentage of PI and Annexin-V double-positive cells. Data are presented as the mean ± SD from three independent experiments; B: cells were stained with Hoechst 33258 and observed under a fluorescence microscope. The percentage of cells with apoptotic bodies was calculated; C: the activation of PARP was evaluated using Western blotting with α-tubulin as a loading control; D: the relative level of cleaved PARP was calculated. Data are presented as the mean ± SD from three independent experiments. ^**^P < 0.01. DCA: dichloroacetate; THP: pirarubicin; PI: propidium iodide

The effect of THP and DCA on apoptosis induction in liver cancer cells was subsequently investigated. As shown in Figure 2A, the percentage of Annexin/PI-positive liver cancer cells in the DCA and THP combination group was significantly higher than that in the DCA or THP alone groups. Consistent with this result, both apoptotic bodies and the apoptosis-related molecule PARP in the DCA and THP combination group were significantly increased [Figure 2B-D]. These results indicated that DCA promoted THP-induced apoptosis in liver cancer cells.

### DCA increases THP-triggered ROS generation in liver cancer cells

**Figure 3:**
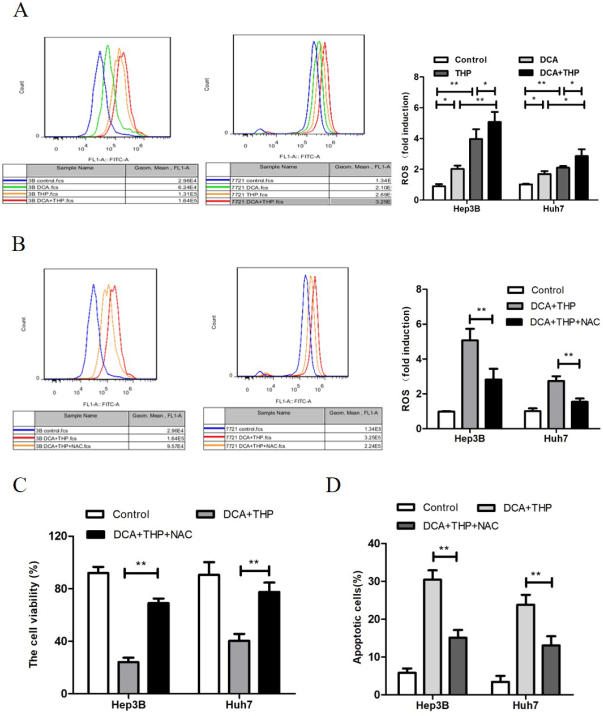
DCA increases THP-triggered ROS generation in liver cancer cells. A: Hep3B and Huh7 cells were treated with DCA (20 mmol/L), THP 300 nmol/L) or a combination of DCA and THP for 24 h. The cells were stained with a CM-H2DCFDA fluorescence probe, and the intensity of DCF fluorescence was measured by flow cytometry. The relative ROS change was presented as the fold change *vs.* the control group; B-D: Hep3B and Huh7 cells were treated with DCA combined with THP or DCA + THP + NAC (10 mmol/L) for 24 h. ROS level was then measured using the CM-H2DCFDA fluorescence probe (B), cell viability was measured using the CCK-8 assay (C) and apoptosis was determined by flow cytometry analysis (D). Data are presented as the mean ± SD from three independent experiments. ^*^P < 0.05, ^**^P < 0.01. DCA: dichloroacetate; THP: pirarubicin; ROS: reactive oxygen species

As high levels of ROS may contribute to increased apoptosis, the effect of THP and DCA on ROS generation was investigated using the CM-H2DCFDA fluorescence probe in the present study. CM-H2DCFDA is a cell-permeable probe used to detect intracellular ROS. It is a derivative of fluorescein with a thiol-reactive chloromethyl group. It allows for covalent binding to intracellular components, permitting even longer retention within the cell. Oxidation of this probe can be detected by monitoring the increase in fluorescence with a flow cytometer, fluorometer, microplate reader, or fluorescence microscope, using excitation sources and filters appropriate for fluorescein. As shown in Figure 3A, the levels of ROS in the THP and DCA only groups were significantly higher than the control group. Moreover, the ROS level in the THP and DCA combination group was significantly higher compared to the THP or DCA only groups (*P* < 0.05). This result suggested that DCA enhanced THP-triggered ROS generation in liver cancer cells. Furthermore, the antioxidants *N*-acetyl-*L*-cysteine (NAC) and dithiothreitol (DTT) significantly attenuated the synergistic effect of DCA and THP on ROS generation [Figure 3B and Supplementary Figure 2], cell viability [Figure 3C and Supplementary Figure 3] and apoptosis induction [Figure 3D and Supplementary Figure 4]. These results indicated that the increase in ROS was responsible for the synergistic effect of DCA and THP in liver cancer cells.

### ROS-JNK signaling pathway plays a key role in the synergistic effect of DCA and THP

**Figure 4:**
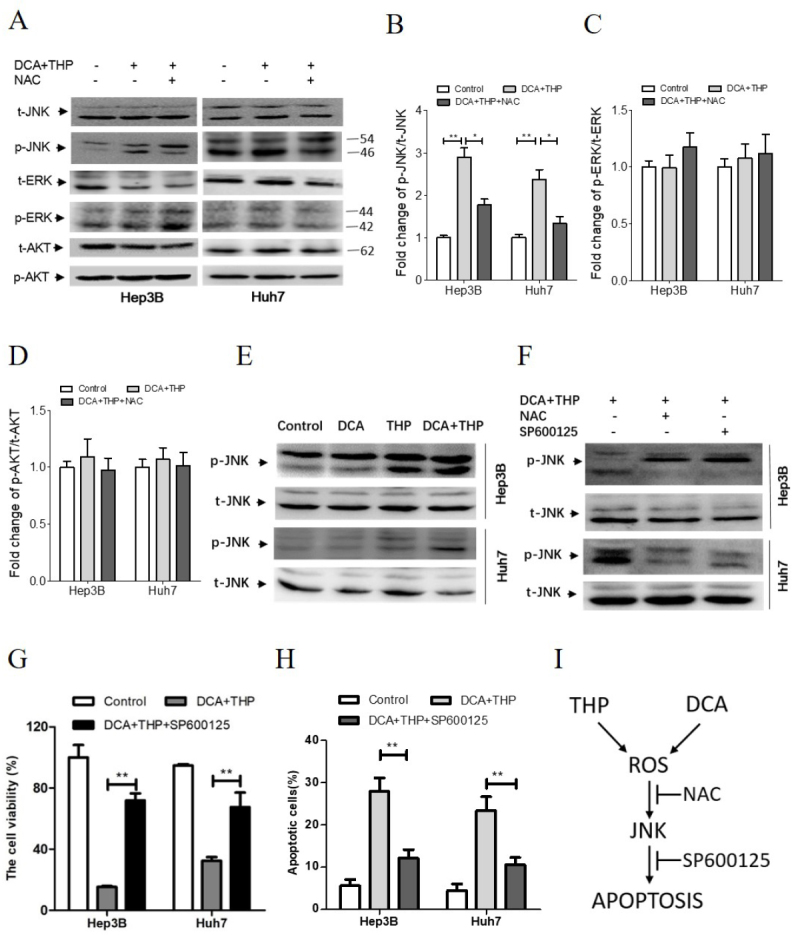
DCA and THP activates the ROS-JNK signaling pathway. A: Hep3B and Huh7 cells were treated with a combination of DCA and THP or DCA + THP + NAC (10 mmol/L) for 24 h and the protein levels of p-JNK, t-JNK, p-AKT, t-AKT, p-ERK and t-ERK were determined by Western blotting; B-D: quantification of the protein levels of p-JNK (B), p-ERK (C) and p-AKT (D). The data were normalized by t-JNK, t-ERK and t-AKT, respectively. The data were expressed as the fold change against the control group and were represented as the mean ± SEM *P < 0.05; **P < 0.01; E: Hep3B and Huh7 cells were treated with DCA (20 mmol/L), THP (300 nmol/L) or a combination of DCA and THP for 24 h, and the protein levels of p-JNK and t-JNK were determined by Western blotting; F-H: Hep3B and Huh7 cells were treated with a combination of DCA and THP or DCA + THP + NAC or DCA + THP + SP600125 (10 µmol/L) for 24 h, where: protein levels of p-JNK and t-JNK were determined by Western blotting (F); cell viability was determined using the CCK-8 assay (G); and apoptosis was calculated by flow cytometry analysis (H); I: schematic illustration for the synergistic effect of DCA and THP in human liver cancer cells. DCA and THP significantly enhanced ROS levels giving rise to JNK activation and apoptosis induction. This synergistic effect of DCA and THP could be attenuated by co-treatment with antioxidant NAC or JNK kinase inhibitor SP600125. Experiments were repeated at least three times. ^**^P < 0.01. DCA: dichloroacetate; THP: pirarubicin; t: total; p: phosphorylated; ROS: reactive oxygen species

Since several signaling pathways could be activated by increased ROS levels, the phosphorylation levels of ERK, AKT and JNK were investigated in the present study. As shown in Figure 4A-D, the phosphorylation of ERK and AKT was not changed following DCA and THP treatment. However, the phosphorylation of JNK was significantly increased. The effects of DCA and THP on the JNK signaling pathway were further investigated. As shown in Figure 4E, the phosphorylation level of JNK was further increased in the THP and DCA combination group, compared with the THP and DCA alone groups. Moreover, the effects of DCA and THP on JNK phosphorylation were suppressed by co-treatment with the antioxidant NAC or DTT or JNK kinase inhibitor SP600125 [Figure 4A, B, F and Supplementary Figure 5]. Furthermore, the effects of DCA and THP on cell viability and apoptosis were inhibited by SP600125 or DTT co-treatment [Figure 4G-H, Supplementary Figures 4 and 6]. These results demonstrated that the ROS-JNK signaling pathway plays a key role in the synergistic effect of DCA and THP.

## Discussion

As a derivative of doxorubicin, THP is widely used to treat hematological malignancies in the clinic^[15]^. Due to a favorable antitumor efficiency with limited side effects, THP has been widely used in TACE and liver metastases^[3,4,16]^. It was shown that THP significantly prolongs the survival of patients with liver cancer, but tumor response is limited because of drug resistance^[17,18]^. Thus, it is urgent to identify cellular signaling pathways targeted to enhance sensitivity of THP. DCA is a small-molecule inhibitor of PDK that has been widely used to treat diabetes mellitus, lipid and lipoprotein disorders, acquired and congenital lactic acidosis for over a decade^[8,19,20]^. This paper shows for the first time that the treatment of human liver cancer cells with DCA and THP significantly enhances ROS levels giving rise to JNK activation and apoptosis induction. DCA significantly enhanced the antitumor effect of THP in liver cancer cells, primarily by promoting THP-triggered apoptosis via the ROS-JNK signaling pathway. This synergistic effect of DCA and THP can be attenuated by antioxidant NAC or JNK kinase inhibitor SP600125 co-treatment. The working model of DCA and THP in liver cancer cells is illustrated in Figure 4I.

Apoptosis induction plays a key role in cancer therapy. Cell fate depends on the balance between pro-survival and pro-apoptotic signaling^[21]^. The level of ROS often affects this balance and therefore regulates cellular activities. Excessive ROS production disrupts this balance and induces apoptosis^[21]^. Previous studies have shown that several drugs, such as oxaliplatin, bevacizumab and salinomycin, can kill cancer cells through ROS induction in liver cancer cells^[22-24]^. A previous study revealed that THP induced apoptosis through hydrogen peroxide generation in HL-60 and HP100 cells^[25]^. The relative ROS level is 159.45% in liver cancer cells treated with DCA alone, 127.28% with Adriamycin alone, and 228.99% in cells treated with a combination of DCA and Adriamycin^[26]^. The combination of DCA and sorafenib also significantly increased ROS levels in liver cancer cells^[11]^. This is consistent with the results described in the present study. The level of ROS in the combination group was significantly higher than that in the DCA alone or THP alone group. ROS elevation induces redox reactions in cancer cells, which increases the antioxidant capacity. However, further stimulation with exogenous oxides increases the level of ROS beyond the threshold level, thus leading to cell death. Interestingly, the ROS level in Hep3B cells was higher than that in Huh7 cells after treatment with DCA and THP. There may be multiple reasons. Hep3B cells have no p53 while Huh7 cells contain mutant p53^[27]^. HCC cells without p53 are more sensitive to intracellular ROS damage^[27]^. Both Hep3B and Huh7 cell lines are well-document models for HBV or HCV study because they are HBV- and HCV-free cells^[28,29]^. However, Huh7 cells are highly susceptible to HCV because they intrinsically express an abundant amount of miR-122, while Hep3B cells show little sensitivity to HCV due to lack of miR-122^[30]^. These differences between Hep3B and Huh7 cells may result in different ROS generation and apoptosis induction upon treatment with DCA and THP. Anyway, DCA significantly enhances the cytotoxicity of THP in liver cancer cells by increasing ROS levels. This provides a theoretical basis for the clinical use of DCA as a chemosensitizer.

The use of DCA for the treatment of solid tumors has been proposed because of its efficacy in reversing the Warburg effect^[31]^. PDK inhibition leads to the reactivation of PDH and oxidative phosphorylation in the mitochondria, thereby decreasing pyruvate and lactate levels^[20]^. Previous studies have demonstrated that DCA increases ROS production, leading to apoptosis of tumor cells^[26,32]^. Consistent with that, the present study showed that DCA alone could trigger ROS generation and significantly enhance THP-induced ROS production in liver cancer cells. Several clinical trials have investigated the safety of chronic oral DCA administration in adults with different tumors, and showed that DCA was generally well-tolerated^[33,34]^. Chronic DCA administration to patients can occasionally lead to mild and symptomatic but reversible elevation of hepatic transaminases^[35]^. However, the chronic administration of up to 25 mg/kg/day oral DCA over several years in patients with primary mitochondrial diseases did not result in significant effects on any indices of hematological, metabolic, renal or hepatic function, including no consistent effects on serum transaminases^[36]^. Therefore, a low dose of DCA could be used as a THP sensitizer in liver cancer cells.

JNK, a type of mitogen-activated protein kinase, is activated by various stimuli, including ROS^[37]^. It is reported that ROS accumulation promotes prolonged activation of JNK by inactivating JNK phosphatases^[38]^. ROS are also an important regulatory factor in the induction of JNK-dependent apoptosis^[39]^. Indeed, JNK phosphorylation and ROS accumulation induced by the combination of DCA and THP can be inhibited by NAC, indicating that the accumulation of ROS results in sustained JNK activation and leads to cell death. Previous studies have demonstrated that induction of apoptosis by activated JNK is essential in modulating the functions of pro- and anti-apoptotic proteins located in the mitochondria^[14,40]^. JNK activation promotes the release of cytochrome c and the cleavage of caspase-3, which result in apoptosis^[41]^. In the present study, this apoptotic activity was found to be blocked by the specific JNK inhibitor SP600125, thus providing further evidence that the mitochondrial apoptotic pathway is regulated by the JNK signaling pathway. We also found that the activation of the ROS-JNK pathway may contribute to major (but not full) cell apoptosis and growth inhibition with DCA and THP treatment. Therefore, NAC or SP600125 co-treatment could not completely protect liver cancer cells from DCA and THP treatment.

In conclusion, the ROS/JNK signaling pathway is an important pathway that induces cell death (including cell necrosis and apoptosis). The present study further revealed that combination treatment with DCA significantly promoted THP-induced ROS elevation and subsequent JNK activation and apoptosis in liver cancer cells.

## Declarations

### Availability of data and materials

The datasets and certain material used and/or analyzed during the present study are available from the corresponding author on reasonable request.

### Copyright

© The Author(s) 2022.
