# Supplementary material for: Dichloroacetate enhances the antitumor effect of pirarubicin via regulating the ROS-JNK signaling pathway in liver cancer cells
Source: Cancer Drug Resist. 2020 Sep 4;3(4):947–58. doi: 10.20517/cdr.2020.32 (PMC8992565; doi:10.20517/cdr.2020.32)
Supplement: Supplementary file 1 [file cdr-3-947-SupplementaryMaterials.pdf]

Supplementary figure legends

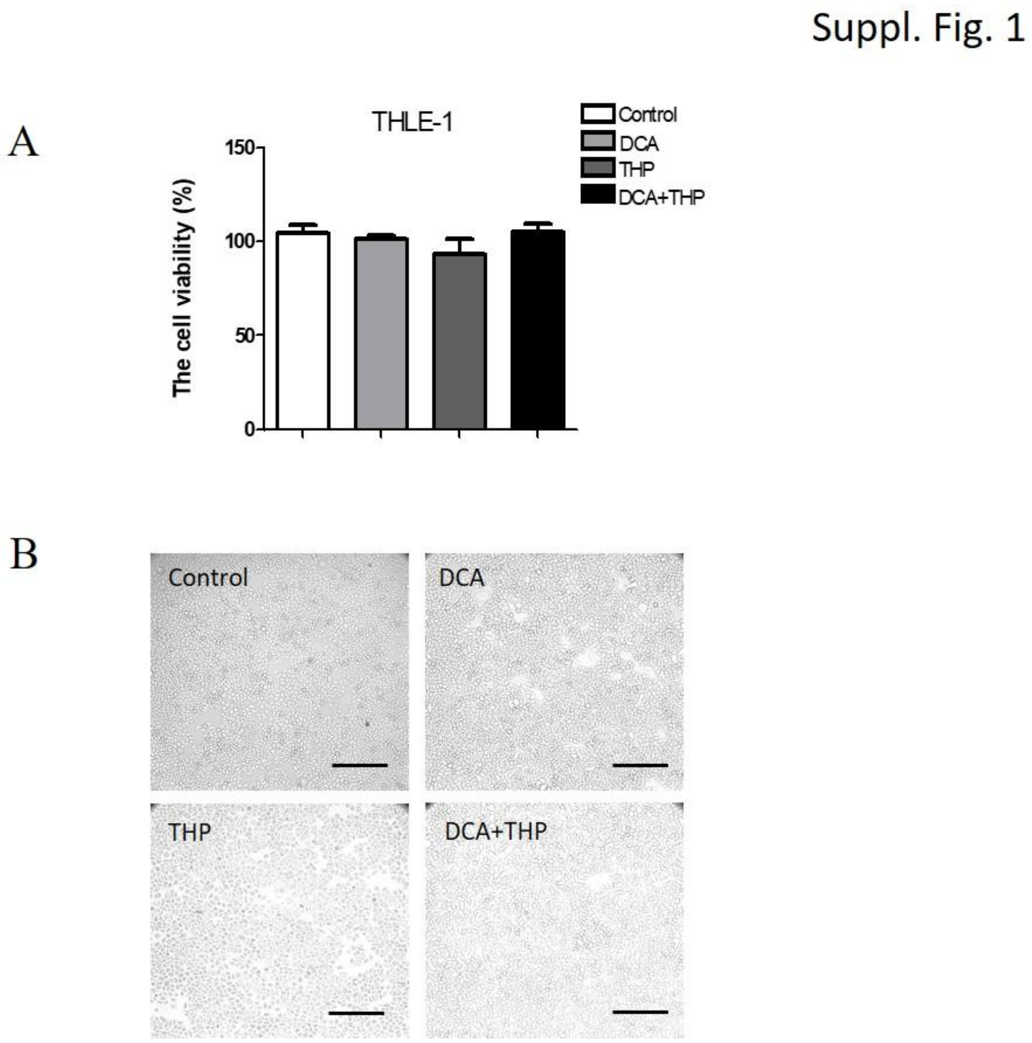

Suppl. Fig. 1

THLE-1 cells were treated with DCA (20 mmol/L), THP (200ng/ml) or DCA combined THP for 24 h. A: Cell viability was measured by CCK-8 kit. B: Cell morphology was observed under a microscope. The experiment is representative of at least three independent experiments.

Suppl. Fig. 2

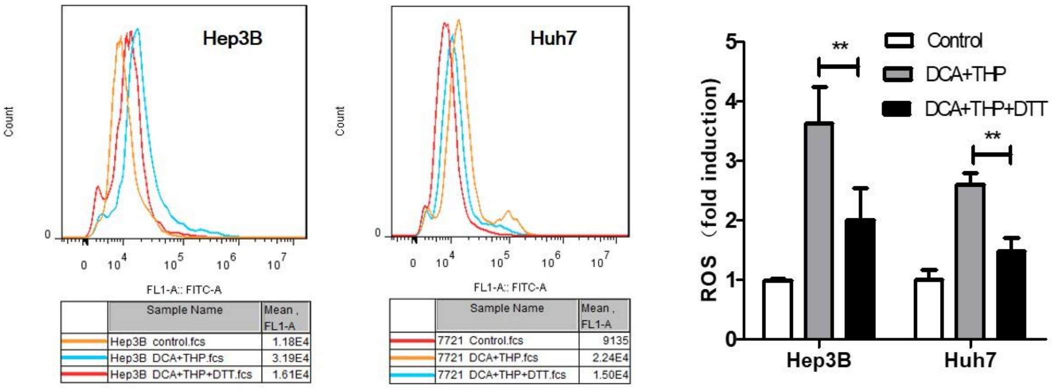

Suppl. Fig. 2

Hep3B and Huh7 cells were treated with DCA combined THP or DCA + THP + DTT (1 mM) for 24 h, then the ROS level was measured by CM-H2DCFDA fluorescent probe. The experiment is representative of at least three independent experiments. **P<0.01. DTT: dithiothreitol

Suppl. Fig. 3

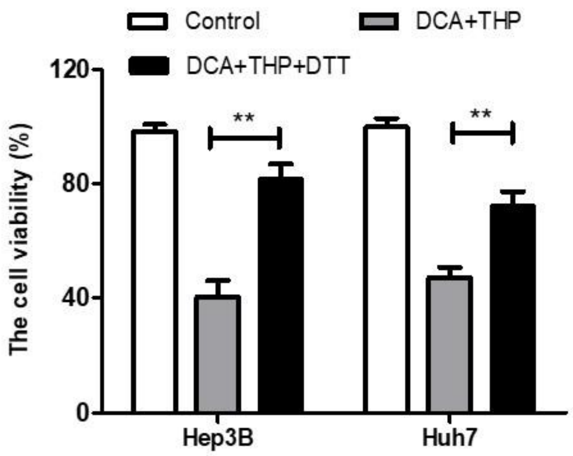

Suppl. Fig. 3

Hep3B and Huh7 cells were treated with DCA combined THP or DCA + THP + DTT

(1 mM) for 24 h, then the cell viability was measured by CCK-8 kit. The experiment is representative of at least three independent experiments. **P<0.01.

Suppl. Fig. 4

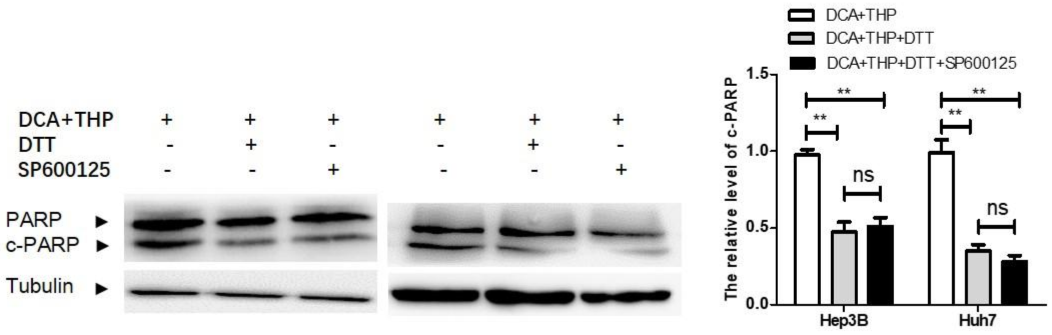

Suppl. Fig. 4

Hep3B and Huh7 cells were treated with DCA + THP or DCA + THP + DTT or DCA + THP + SP600125 (10 μM) for 24 h, then the activation of PARP was evaluated using western blotting. And the relative level of cleaved PARP was calculated. Data represent the mean value and SD from 3 independent experiments. **p<0.01.

Suppl. Fig. 5

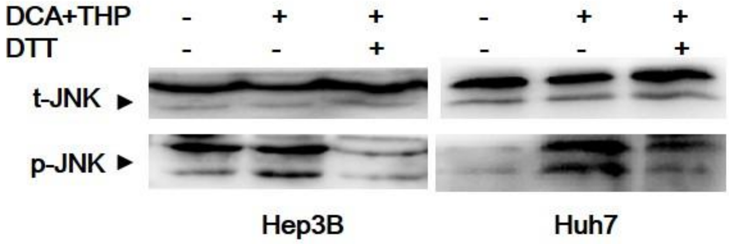

Suppl. Fig. 5

Hep3B and Huh7 cells were treated with DCA combined THP or DCA + THP + DTT (1 mM) for 24 h, then the protein levels of p-JNK and t-JNK were detected by

western blotting.

Suppl. Fig. 6

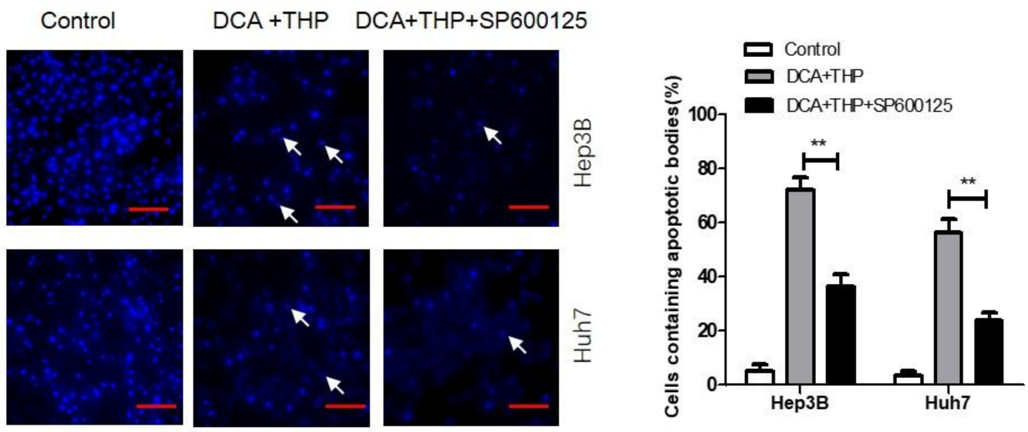

Suppl. Fig. 6

Hep3B and Huh7 cells were treated with DCA + THP or DCA + THP + DTT or DCA + THP + SP600125 (10 μM) for 24 h, then cells were stained with Hoechst 33258 and observed under a fluorescence microscope. And the percentage of cells with apoptotic bodies was calculated. Data represent the mean value and SD from 3 independent experiments. **p<0.01.
